# Comparative effects of oral nutritional supplementation vs. nutritional education on appetite and weight in older adults with anorexia of aging: a 12-week non-randomized controlled trial

**DOI:** 10.3389/fnut.2025.1606008

**Published:** 2025-05-20

**Authors:** Gaojie Feng, Chen Liu, Xiaohong Sun, Xiaohong Liu, Fei Lu, Yuanyuan Li, Yaru Zhou

**Affiliations:** Department of Geriatrics, Peking Union Medical College, Chinese Academy of Medical Sciences, Peking Union Medical College Hospital, Beijing, China

**Keywords:** clinical trial, community-dwelling older adults, oral nutritional supplement (ONS), SNAQ, weight

## Abstract

**Background:**

With global aging, diet education and oral nutritional supplements (ONS) are recognized for improving nutrition and appetite in older adults, yet evidence on anorexia of aging (AA) mechanisms and interventions remains limited in China. This study aimed to evaluate diet education and ONS efficacy for AA in Chinese community-dwelling older adults.

**Methods:**

In an open-label, non-randomized controlled trial, 64 eligible participants were allocated to an ONS group (supplementation) or a diet education group. The Simplified Nutritional Appetite Questionnaire (SNAQ) assessed AA, with follow-ups at weeks 2, 4, 8, and 12. Primary outcomes were SNAQ improvements; secondary outcomes included weight, grip strength, nutritional status (BMI, MNA-SF), cognition (MMSE), mobility (SPPB), mental health (GDS15), and quality of life (EQ-5D).

**Results:**

Younger, non-solo-living, and malnourished participants preferred ONS intervention (*p* < 0.05). Both groups showed increased SNAQ scores versus baseline (counseling: weeks 4/8/12; ONS: weeks 2/4/8/12), with ONS achieving significantly greater improvement at week 2 (*p* < 0.05). Weight remained unchanged in both groups (*p* > 0.05). Diet education increased grip strength at week 12 (*p* < 0.05), while no significant improvements occurred in BMI, cognition, mobility, or quality of life.

**Conclusion:**

Both ONS and diet education alleviated AA over 12 weeks, but ONS demonstrated earlier efficacy (significant SNAQ improvement by week 2). However, ONS did not enhance weight, physical function, or cognitive outcomes.

**Trial registration:**

Approved by Peking Union Medical College Hospital Ethics Committee (I-23PJ661), registered at Chinese Clinical Trial Registry (MR-11-23-023104).

## 1 Introduction

Anorexia of aging (AA) refers to age-related decreased food intake resulting from physiological, pathological, and psychological factors, characterized by appetite decline and insufficient nutrient intake (1). First conceptualized in 1980, AA has yet to achieve international diagnostic consensus. This condition arises from multifactorial interactions including senescence, functional impairment, environmental influences, chronic diseases, and polypharmacy. These factors collectively contribute to multisystem dysregulation involving oral, digestive, sensory, and neuroendocrine functions, leading to clinically significant outcomes such as weight loss (2), malnutrition (3, 4), sarcopenia (5–9), frailty (10, 11), disability, and increased mortality (4, 12). With accelerating global population aging, AA has emerged as a critical geriatric health concern. However, limited awareness, heterogeneous diagnostic criteria, and inadequate screening tools hinder accurate epidemiological assessment. With higher rates observed in institutionalized populations compared to community settings (13), international studies reported AA prevalence ranging from 6.9 to 30% in community-dwelling older adults (11, 14, 15). This underscores the importance of community-based early intervention strategies. Despite this growing burden, China faces systemic challenges in AA recognition and management, evidenced by limited epidemiological data. Our 2023 Beijing community screening (age ≥60 years) identified AA prevalence at 22.1% (16), corroborated by a 2024 Shandong province study reporting 21.7% prevalence among 2,144 rural/urban residents (17). This highlights the urgent need for systematic screening and targeted interventions for AA in China’s aging population.

Regarding interventions with AA, a 2019 UK systematic review (18) synthesized evidence from 18 studies evaluating AA management strategies, including nutritional education, exercise programs, flavor enhancement, dietary modification, oral nutritional supplementation (ONS), and pharmacological interventions. Key findings indicated that flavor enhancement and dietary diversification effectively stimulated hunger perception. Specifically, oleic acid-enriched ONS demonstrated potential appetite improvement, while pharmacological agents showed mixed efficacy with notable adverse effects. While international studies suggest ONS improve nutritional status and appetite in older adults (18–20), evidence in Chinese populations remains scarce. Previous trials predominantly relied on subjective appetite assessments, lacking standardized longitudinal monitoring. Our research team has already validated that the Chinese version of the Simplified Nutritional Appetite Questionnaire (SNAQ) has good reliability and validity among community-dwelling older adults with AA (16). This study addresses these gaps by evaluating the effects of ONS versus diet education using the validated SNAQ and multidimensional comprehensive geriatric assessments. This trial aims to evaluate the differential effects of personalized ONS interventions versus diet education in community-dwelling older adults with AA in improving appetite, functional status, and comprehensive health outcomes among Chinese community-dwelling AA patients.

## 2 Materials and methods

### 2.1 Study design

This study adopted a 3-month preference-adaptive trial design, a subtype of pragmatic non-randomized controlled trials among community-dwelling older adults (aged 65 years and older) with AA. Participants were empowered to choose either the ONS arm or the nutrition education arm through structured shared decision-making. This approach explicitly accommodated three critical real-world constraints: (1) financial burden mitigation (as ONS required out-of-pocket costs), (2) prevention of dietary intolerance risks (e.g., lactose allergy), and (3) respect for cultural preferences regarding commercial nutritional products. Allocation transparency was ensured through an audited decision aid tool documenting choice rationales. Recruitment occurred between May 1, 2023 and October 1, 2024 in Beijing. This study has been approved by the Peking Union Medical College Hospital Ethics Committee (I-23PJ661) and registered at the Chinese Clinical Trial Registry (MR-11-23-023104). All participants were provided informed consent prior to enrollment.

**Figure 1:**
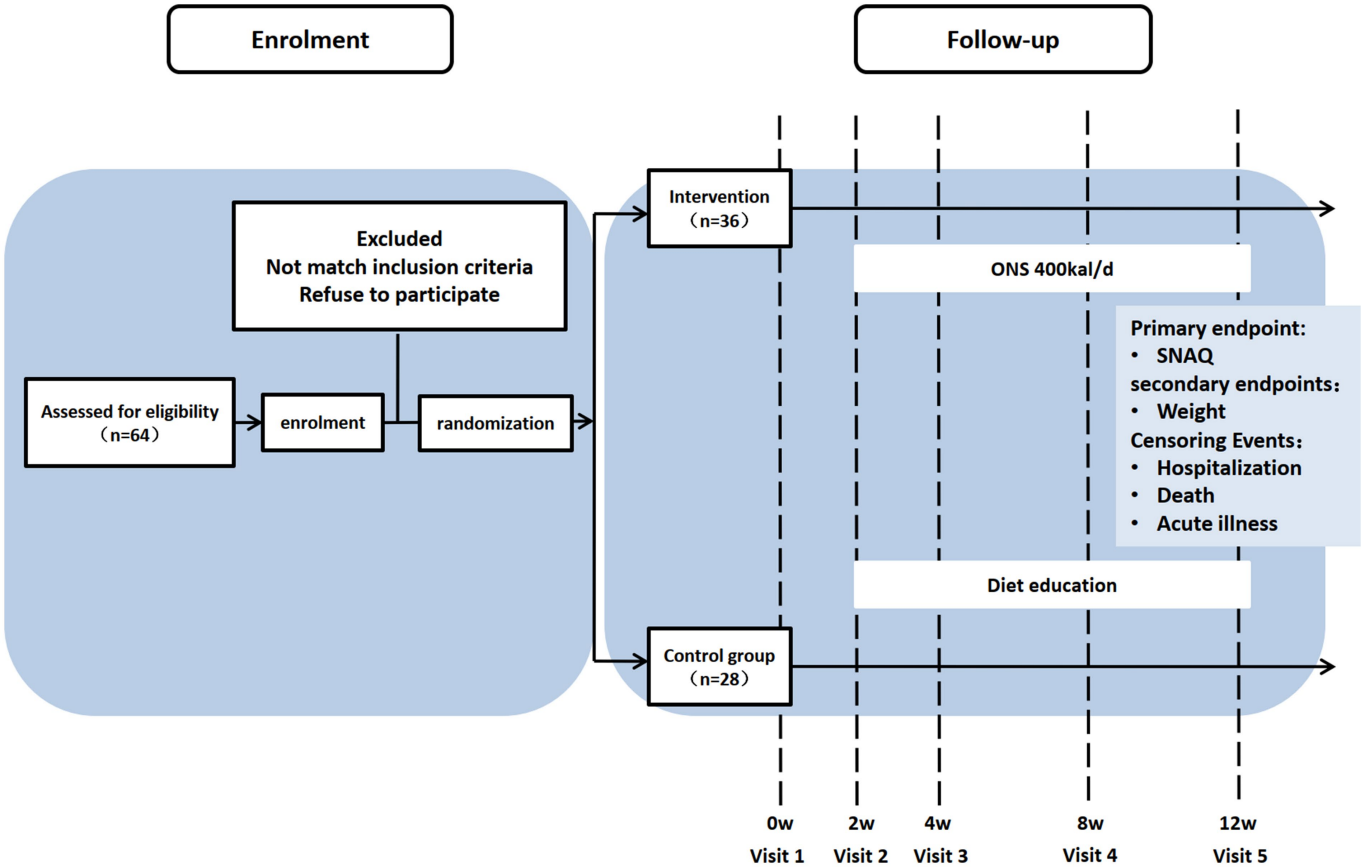
Participant flow diagram.

The flow diagram was created using the CONSORT PRO template (Evidence Partners, Canada) (Figure 1).

### 2.2 Recruitment, inclusion and exclusion

From May 2023 to October 2024, community-dwelling older adults with AA were recruited through poster advertisements and health lectures conducted in local communities. Potential participants contacted geriatricians via telephone and underwent face-to-face eligibility assessments. Inclusion criteria required individuals to be aged ≥65 years, score ≤14 on SNAQ, exhibit intact communication abilities, maintain ambulatory capacity (assistive devices permitted), and demonstrate stable management of chronic conditions. Exclusion criteria comprised bedridden status, dementia diagnosis, advanced malignancies, life expectancy <6 months, and failure to provide written informed consent.

### 2.3 Intervention method

Participants were allocated into two study groups and committed to weekly self-monitoring of body weight under standardized conditions: morning post-void measurements while wearing light clothing, after emptying the bowels, using digital scales calibrated to 0.1-kilogram precision.

#### 2.3.1 Control group

The control group received diet education from geriatricians, which included standardized written guidelines outlining key nutritional recommendations: daily energy intake targets of 30 kilocalories per kilogram of body weight, protein distribution emphasizing 1 g per kilogram per day through high-quality sources such as poultry, fish, eggs, legumes, and dairy products, hydration requirements of 30 milliliters per kilogram daily, and a minimum daily intake of 500 g of fresh vegetables and fruits. These evidence-based nutritional principles were reinforced through printed educational materials distributed during baseline consultations.

#### 2.3.2 ONS group

Participants in the ONS group collaboratively developed personalized nutritional supplementation plans with geriatricians, with individualized protocols based on diabetes status: non-diabetic patients received Ensure^®^ Vanilla (three scoops dissolved in 100 mL water three to four times daily [TID ~ QID]), while those with diabetes were prescribed Glucerna^®^ (3.5 scoops reconstituted in 100 mL water TID/QID). All supplements should be administered at intervals separated from meal times, reconstituted with lukewarm water (30–40°C), and consumed in small, deliberate sips.

Our pre-study literature review found no significant differences in their macronutrient profiles (protein: 15.9 vs.19.51 g/100 g; fat: 15.9 g vs.15.9 g/100 g; carbohydrate: 60.7 g vs. 41.15 g/100 g) or energy density (1.0–1.2 kcal/mL). We recommended daily energy intake standardized to 397–530 kcal/day for all participants in the ONS group.

### 2.4 Measures and follow-up

**Figure 2:**
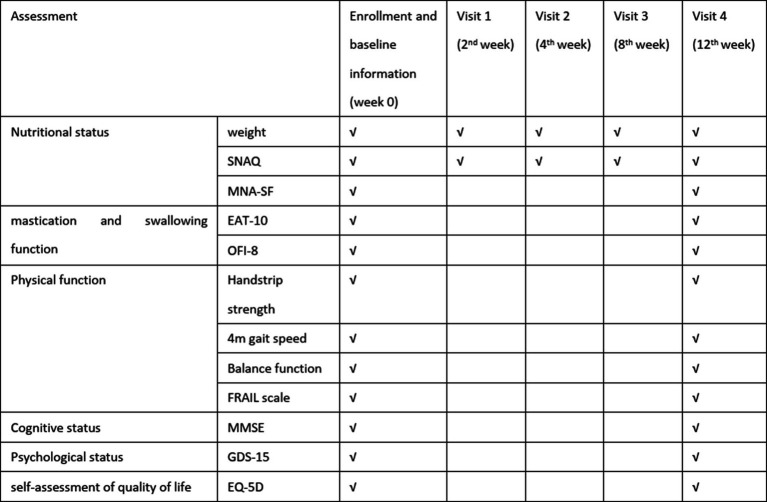
Schedule of assessment.

All baseline and final assessments were conducted through face-to-face evaluations by geriatricians, while the 3-month follow-up data (SNAQ and self-weight monitor in weeks 2, 4, and 8) were collected by the same physicians through structured telephone interviews or WeChat text-based communication to ensure consistency in data collection methodology (schedule of assessment as shown in Figure 2).

#### 2.4.1 Data collection baseline and final assessments

A researcher-developed questionnaire captured demographic characteristics [gender, age, living alone or not, weight, drug number and the Charlson Comorbidity index (CCI)]. Baseline physical function was evaluated through grip strength (Jamar dynamometer), 4-meter gait speed, five-times sit-to-stand test, and static balance assessments. Multidimensional health metrics included (as shown in Figure 2):

- Appetite: SNAQ
- Nutritional status: Mini-Nutritional Assessment Short-Form (MNA-SF)
- Frailty: FRAIL scale
- Quality of life: EuroQol Five-Dimensional Questionnaire (EQ-5D)
- Psychological status: 15-item Geriatric Depression Scale (GDS-15)
- Swallowing function: 10-item Eating Assessment Tool (EAT-10)
- Oral health: 8-item Oral Frailty Index (OFI-8)
- Mobility: short physical performance battery (SPPB)

All instruments were administered by trained geriatricians using standardized protocols, with anthropometric measurements conducted using calibrated devices.

#### 2.4.2 Follow-up

The primary outcome was the change in SNAQ scores at 12 weeks. Secondary outcomes included anthropometric measurements (body weight), physical function indicators (grip strength, SPPB), functional status, nutritional status assessed by Mini-Nutritional Assessment Short-Form (MNA-SF), frailty severity measured using FRAIL scale, cognitive function evaluated through Mini-Mental State Examination (MMSE), psychological status via 15-item Geriatric Depression Scale (GDS-15), and quality of life using EuroQol Five-Dimensional Questionnaire (EQ-5D).

### 2.5 Statistics

#### 2.5.1 Sample size

The sample size estimation was based on appetite improvement as the primary endpoint. Referring to a previous randomized controlled trial by Faxén-Irving and Cederholm et al. (21) demonstrating significant appetite enhancement through ONS intervention (*n* = 51 completers), we calculated the required sample size using visual analog scale (VAS) appetite scores (0–10 scale). A clinically meaningful difference of 3 points on VAS (corresponding to ≥1-point improvement on SNAQ Question 1) was set as the threshold.

The sample size was determined for a two-arm parallel-group superiority trial with continuous outcomes (appetite improvement as the primary endpoint). Using the formula for equal group sizes in a two-sided test:


$$n=\frac{{\left(Z_{1-\alpha /2}+Z_{1-\beta }\right)}^{2}\times (\sigma_{1}^{2}+\sigma_{2}^{2})}{\delta^{2}}$$

Where:

*Z*_1 − α/2_ = 1.96, *Z*_1 − α/2_ = 1.96 (α = 0.05, two-tailed).

*Z*_1 − β_ = 1.28, *Z*_1 − β_ = 1.28 (β = 0.10, 90% power).

σ_1_ and σ_2_ = Standard deviations from the Gerd trial reference population.

δ = Clinically meaningful intergroup difference.

The calculated minimum sample size per group was 19. To account for a 20% anticipated dropout rate, the final recruitment target was set to 23 participants per group (total *N* = 46).

#### 2.5.2 Statistical analysis

All analyses will be performed using SPSS 22.0 (IBM Corp.) with two-tailed significance (*α* = 0.05). Continuous variables will be presented as Mean ± SD (normally distributed) or median [IQR] (non-normal) and categorical variables will be presented as frequency (%). Baseline characteristics will be compared using Independent t-tests/Mann–Whitney *U* tests for continuous variables and Chi-square tests for categorical variables. Primary analysis will employ using analysis of covariance (ANCOVA) to assess between-group differences in SNAQ changes (baseline-adjusted) and Linear mixed-effects models for longitudinal analysis of repeated measures (SNAQ and weight). In addition, Cohen’s *d* was used to quantify standardized mean differences for intervention effects.

## 3 Results

### 3.1 Baseline characteristics

A total of 64 participants were enrolled, 28 in the diet education group (control) and 36 in the ONS intervention group. As shown in Table 1, the intervention group was significantly younger (*p* = 0.008), had lower rates of living alone (*p* = 0.048), and exhibited poorer baseline nutritional status as assessed by MNA-SF (*p* = 0.015). No significant between-group differences were observed in gender distribution, drug number, comorbidities (CCI), and functional and physical parameters (*p* > 0.05).

**Table 1:** Baseline sociodemographic and clinical characteristics of community-dwelling older adults with AA.

Variables	Control (*n* = 28)	ONS Intervention (*n* = 36)	*t/χ^2^/Z*	*p-*value
General characteristics				
Age (years)	82.17 ± 8.33	75.92 ± 8.91	2.73	0.008
Male, *n*(%)	11 (40.7)	15 (40.5)	0.000	0.987
Living alone, *n*(%)	8 (33.3)	2 (7.4)	3.898	0.048
Drug number, median (Q25, Q75)	7 (4,11.5)	5.5 (1.25,9)	1.213	0.234
CCI	1.43 ± 1.62	1.35 ± 1.86	0.175	0.862
Physical function				
HGS, kg	20.64 ± 8.47	20.78 ± 7.07	−0.073	0.942
BMI, kg/m^2^	21.79 ± 5.76	20.35 ± 4.35	1.134	0.261
SPPB	8.6 ± 3.4	8.2 ± 3.3	0.456	0.651
Comprehensive geriatric assessment, CGA				
FRAIL	1.22 ± 1.28	1.88 ± 1.36	−1.906	0.062
EAT-10	1.52 ± 2.06	1.74 ± 2.53	−0.363	0.718
OFI-8	5.84 ± 2.39	5.14 ± 2.20	1.167	0.248
MMSE	27.74 ± 2.80	26.08 ± 5.05	1.660	0.102
GDS15	3.35 ± 2.38	3.79 ± 3.50	−0.561	0.577
EQ-5D	7.86 ± 4.55	9.34 ± 5.11	−1.203	0.234
MNA-SF	11.04 ± 2.80	9.14 ± 3.08	2.512	0.015
SNAQ-baseline	12.59 ± 1.50	12.06 ± 1.88	1.220	0.227

BMI, body mass index; CCI, Charlson Comorbidity Index; HGS, Handgrip strength; MNA-SF, Mini Nutritional Assessment Short-Form; SPPB, short physical performance battery.

Data presented as mean ± SD or frequency (%).

### 3.2 Primary outcome: SNAQ

**Figure 3:**
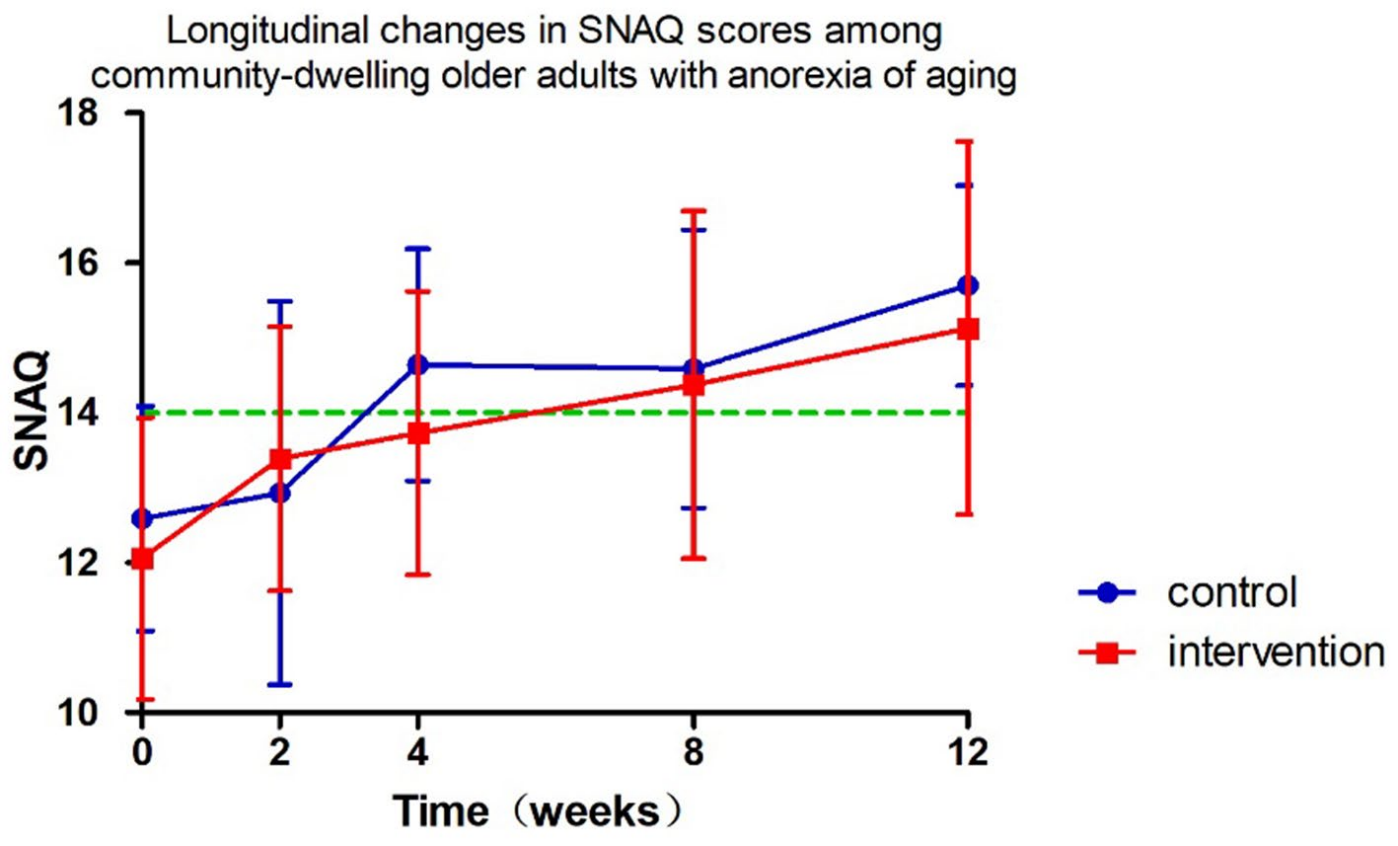
Follow-up changes in SNAQ in community-dwelling elderly with anorexia of aging.

The longitudinal profiles of SNAQ scores (baseline and follow-up) for both groups are displayed in Figure 3, with specific values documented in Tables 2, 3. During the 12-week follow-up period, SNAQ scores increased compared to baseline in all participants. This study found that SNAQ measurements in both the ONS supplementation and diet education groups showed statistically significant improvements compared to baseline at weeks 4, 8, and 12. At week 2, the ONS group exhibited a statistically significant increase in SNAQ scores relative to baseline (*p* < 0.05), whereas no such significance was observed in the dietary education group (Table 2).

**Table 2:** Longitudinal changes in SNAQ in community-dwelling older people with anorexia of aging.

SNAQ difference	Control	*t/Z*	*P*-value	Intervention	*t/Z*	*P*-value
Change-2nd week	0.5 (2.75)^#^	−0.217	0.831	2.0 (2.0)^#^	−7.173	<0.001
Change-4th week	2.0 (3.0)^#^	−4.006	0.001	1.42 ± 2.12^*^	3.423	0.002
Change-8th week	1.63 ± 1.90^*^	2.968	0.013	2.04 ± 2.81^*^	3.566	0.002
Change-12th week	3.0 (4.0)^#^	−4.353	0.001	2.52 ± 2.71^*^	4.460	<0.001

^*^Indicates normal distribution, paired-samples *t*-test is used; ^#^indicates non-normal distribution, non-parametric tests for two related samples are used.

**Table 3:** SNAQ score changes: group comparisons at weeks 2, 4, 8, 12.

Group	Control	Intervention	*t*	*P*	Cohen’s *d*	P^*^
ΔSNAQ in 2 weeks	−0.07 ± 2.34	2.04 ± 1.45	−2.872	0.004	1.191	0.032
ΔSNAQ in 4 weeks	1.57 ± 1.55	1.42 ± 2.12	0.230	0.819	0.077	0.092
ΔSNAQ in 8 weeks	1.63 ± 1.90	2.04 ± 2.80	−0.463	0.646	0.263	0.406
ΔSNAQ in 12 weeks	2.18 ± 1.66	2.52 ± 2.71	−0.381	0.706	0.140	0.518

^*^After adjusting for baseline SNAQ scores, age, and gender using ANCOVA.

The intervention group showed superior SNAQ score improvement at week 2 compared to diet education (*p* = 0.032) after controlling for baseline scores, age, and gender using ANCOVA. Baseline SNAQ score had a significant negative impact on the improvement magnitude (*p* = 0.040), while age and gender had no significant effects (*p* > 0.05). After adjusting for baseline SNAQ score, age, and gender, no significant differences were found in the ΔSNAQ score between the two groups at Week 4 (*p* = 0.092), Week 8 (*p* = 0.406), and Week 12 (*p* = 0.518). Longitudinal analysis of SNAQ scores via linear mixed-effects models, adjusted for age, gender, baseline SNAQ scores, timepoints (weeks 0, 2, 4, 8, 12), group assignment, and group-by-time interaction, revealed the following (Table 4):

- Group-by-time interaction effect: non-significant (*β* = −0.031, 95% CI: −0.140 to 0.077, *p* = 0.571), indicating no statistically significant difference in the rate of SNAQ improvement between the ONS and diet education interventions.
- Time main effect: significant (*β* = 0.218 per week, *p* < 0.001), suggesting an overall improvement in SNAQ scores over time across all participants.
- Baseline SNAQ score: each 1-point increase in baseline SNAQ was associated with a 0.566-point increase in follow-up scores (*β* = 0.566, *p* < 0.001).
- Age: a negative correlation was observed, with each additional year of age linked to a 0.032-point decrease in SNAQ scores (*β* = −0.032, *p* = 0.101).
- Gender: no significant effect on SNAQ scores (*β* = 0.063, *p* = 0.855).

**Table 4:** Fixed-effects analysis of SNAQ using linear mixed-effects models.

Parameters	*β*	*t*	*P*	95%CI	
				Lower limit	Upper limit
Intercept	8.215	3.706	<0.001	3.785	12.644
Weeks	0.218	4.811	<0.001	0.128	0.307
Age	−0.032	−1.666	0.101	−0.071	0.006
SNAQ baseline	0.566	5.731	<0.001	0.369	0.763
Intervention	0.084	0.197	0.844	−0.760	0.928
Female	0.063	0.184	0.855	0.617	0.742
Intervention*week	−0.031	−0.567	0.571	−0.140	0.077

The control group and males were set as the reference group.

### 3.3 Secondary outcomes

#### 3.3.1 Weight

**Figure 4:**
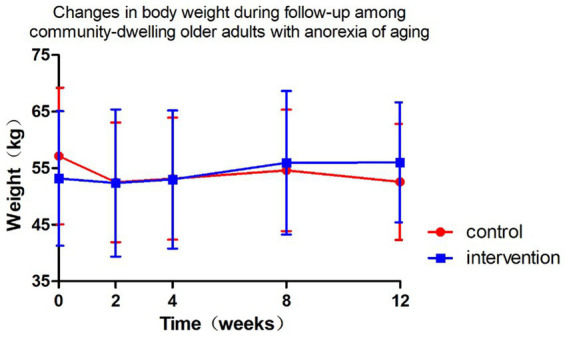
Changes in body weight during follow-up among community-dwelling older adults with anorexia of aging.

Figure 4 and Tables 5, 6 present the body weight changes, revealing no statistically significant within-group or between-group differences. Comparisons of weight changes at weeks 2, 4, 8, and 12 relative to baseline between the intervention and control groups showed no statistically significant differences in ANCOVA analyses adjusted for baseline weight, age, and gender. In the control group, weight at week 2 and in the intervention group at weeks 2, 4, 8, and 12 showed increases compared to baseline, but these differences were not statistically significant (Table 6).

**Table 5:** Longitudinal changes in weight in community-dwelling older people with anorexia of aging.

Weight difference	Control	*t/Z*	*P-*value	Intervention	*t/Z*	*P-*value
Change-2nd week	0.75 (1.74)^#^	−0.721	0.483	0.26 ± 1.34^*^	0.990	0.332
Change-4th week	0.0 (1.35)^#^	0.314	0.758	0.29 ± 1.35^*^	1.065	0.298
Change-8th week	0.01 ± 2.05^*^	0.021	0.984	1.29 ± 4.42^*^	1.464	0.156
Change-12th week	1.1 (5.25)^#^	0.115	0.911	0.7 (2.55)^#^	−0.930	0.364

^*^Indicates normal distribution, paired-samples *t*-test is used; ^#^indicates non-normal distribution, non-parametric tests for two related samples are used.

**Table 6:** Weight changes: group comparisons at weeks 2, 4, 8, 12.

Group	Control	Intervention	*t*	*P*	Cohen *d*	P^*^
Δweight in 2 weeks	0.39 ± 1.98	0.26 ± 1.33	0.233	0.817	0.021	0.872
Δweight in 4 weeks	−0.13 ± 1.67	0.29 ± 1.35	−0.888	0.380	0.283	0.526
Δweight in 8 weeks	0.01 ± 2.05	1.29 ± 4.42	−0.914	0.367	0.442	0.242
Δweight in 12 weeks	−0.07 ± 3.21	0.48 ± 2.38	−0.522	0.606	0.446	0.260

^*^After adjusting for baseline weight, age, and gender using analysis of covariance (ANCOVA).

Longitudinal analysis of weight using linear mixed-effects models, adjusted for age, gender, baseline weight, timepoints (weeks 0, 2, 4, 8, 12), group assignment, and group-by-time interaction, revealed the following (Table 7):

- Baseline weight: for every 1 kg increase in baseline weight, follow-up weight was 0.942 kg higher (*β* = 0.942, *p* < 0.001).
- Gender: females had 1.181 kg lower baseline weight than males (*β* = −1.181, *p* = 0.022).
- Group assignment: no baseline weight difference between intervention and control groups (*β* = −0.075, *p* = 0.883).
- Age: no significant effect on weight (*β* = −0.013, *p* = 0.583).
- Time effect in controls: weight in the control group did not change significantly over time (*β* = −0.010/week, *p* = 0.862).
- Intervention effect: the intervention group gained 0.080 kg more per week than the control group, but this difference was not statistically significant (*β* = 0.080, *p* = 0.232).

**Table 7:** Fixed-effects analysis of SNAQ using linear mixed-effects models.

Parameters	*β*	*t*	*P*	95%CI	
				Lower limit	Upper limit
Intercept	5.034	2.137	0.038	0.284	9.784
Week	−0.010	−0.174	0.862	−0.119	0.100
Age	−0.013	−0.552	0.583	−0.061	0.035
Weight baseline	0.942	46.546	<0.001	0.901	0.982
Intervention	−0.075	−0.148	0.883	−1.081	0.932
Female	−1.181	−2.386	0.022	−2.179	−0.183
Intervention*week	0.080	1.199	0.232	−0.052	0.213

The control group and males were set as the reference group.

#### 3.3.2 Comprehensive geriatric assessment

At 12 weeks, grip strength significantly increased in the control group (*p* < 0.05), while the intervention group showed a non-significant upward trend. No significant improvements were observed in nutritional parameters (BMI, grip strength, MNA-SF), cognitive function (MMSE), mobility (SPPB), psychological status (GDS-15), or quality of life (EQ-5D) (Table 8).

**Table 8:** The changes in the comprehensive geriatric assessment of community-dwelling older adults with AA at week 12 compared with baseline.

CGA	Control	*t*	*P*	Intervention	*t*	*P*
Change-FRAIL	−0.50 ± 1.45	1.198	0.256	−0.47 ± 1.07	−1.710	0.087
Change-MMSE	−0.57 ± 1.90	0.795	0.457	0.35 ± 2.03	−0.540	0.589
Change-MNA-SF	0.50 ± 1.87	−0.655	0.542	1.06 ± 2.69	−1.665	0.114
Change-GDS-15	−0.14 ± 2.12	0.179	0.864	0.00 ± 3.01	0.000	1.000
Change-EQ-5D	2.14 ± 4.74	−1.196	0.277	−0.79 ± 2.32	−1.466	0.143
Change-grip strength	2.73 ± 2.90	−2.486	0.047	0.93 ± 2.29	−1.763	0.095
Change-SPPB	1.00 ± 1.73	−1.528	0.177	−0.158 ± 1.46	0.470	0.644
Change-BMI	−0.15 ± 1.35	0.298	0.777	0.29 ± 0.72	−1.759	0.096

## 4 Discussion

As the first non-randomized controlled trial in China investigating the effects of ONS on AA, this study yielded clinically significant findings. The data demonstrated that community-dwelling older adults who were relatively younger, cohabiting with family members, and at higher nutritional risk showed greater preference for ONS intervention, whereas their older, solitary-living counterparts with lower nutritional risk tended to opt for nutritional education (*p* < 0.05).

Both interventions significantly improved appetite. The ONS group exhibited rapid SNAQ score elevation as early as week 2 (*p* < 0.05), confirming its advantage in acute-phase management. Although the nutrition education group showed slower onset, it achieved comparable improvement levels to ONS after week 4. Notably, mixed-effect models revealed no statistically significant difference in improvement trends between groups (interaction effect *p* = 0.571), with a mean weekly improvement of 0.218 points suggesting potential natural recovery or education effects. The substantial prognostic impact of baseline SNAQ scores (*β* = 0.57) outweighed that of intervention modalities, providing crucial evidence for future stratified interventions.

Current evidence remains inconclusive regarding ONS mechanisms. While theoretically high-nutrient-density formulations can rapidly correct energy-protein deficiencies (22), actual efficacy depends on multiple factors including baseline nutritional status and comorbidities (23–26). Importantly, combined exercise intervention may represent a future research direction, as existing meta-analyses indicate standalone ONS has limited isolated effects on appetite despite improving weight-related parameters (19). The current study’s inability to assess post-intervention sustainability due to observation period limitations warrants investigation in future research.

The study findings demonstrated no statistically significant weight changes in community-dwelling older adults with AA during the 12-week intervention period, regardless of receiving ONS or nutritional education (*P* > 0.05). Although the ONS group showed a marginal weight gain tendency (additional 0.08 kg/week), this difference lacked both statistical significance (*p* = 0.232) and clinical relevance (merely 0.96 kg accumulated difference over 3 months). Notably, baseline weight substantially predicted follow-up weight (*β* = 0.94), and female participants generally maintained lower body weight than males (*β* = −1.18 kg, *p* = 0.022), suggesting the necessity for more aggressive interventions targeting low-weight populations.

Existing evidence regarding ONS effects on weight remains controversial. While some studies support weight-promoting effects, these may be limited to specific populations [e.g., malnourished individuals (20)] or particular body components [e.g., fat-free mass (27) or fat mass (28, 29)]. The absent weight improvement in our study may stem from unquantified energy intake between groups and age-related eating behavior modifications characterized by diminished hunger and enhanced satiety. Importantly, combined exercise interventions [particularly HMB-fortified ONS with exercise (30)] may yield superior outcomes through body composition improvements (31).

The most significant implication lies in recognizing that weight change may not represent an optimal evaluation metric for AA management. While appetite significantly improved after 12-week interventions, this benefit did not translate into measurable weight alterations (32), potentially reflecting unique energy metabolism patterns in older adults. Future research should incorporate body composition analysis and consider integrating exercise training to develop more precise nutritional strategies.

The study revealed a distinct pattern of grip strength changes after 12-week interventions: the nutrition education group demonstrated significant improvement (Δ = 2.73 ± 2.9 kg, *p* = 0.047), while the ONS group showed non-significant enhancement (Δ = 0.93 ± 2.29 kg, *p* = 0.095). This discrepancy with previous findings may be explained by several factors: First, formulation differences likely played a pivotal role. The ONS used in this trial lacked whey protein or HMB – bioactive components proven to improve muscle function (33). Importantly, the ONS group’s poorer baseline nutritional status (lower MNA-SF scores) suggests that more severely malnourished older adults may require longer intervention periods to manifest strength gains. Second, multiple mechanisms could contribute to the education group’s improvement. Beyond potential nutritional amelioration, modified dietary behaviors or spontaneous physical activity increases might underlie this effect. However, study design limitations precluded systematic monitoring of energy intake and exercise, necessitating future verification through diet-exercise logs. Finally, relevant studies in hospitalized older patients indicate grip strength may remain stable even over 6-month observation (34). This implies that short-term nutritional interventions may have limited impact on muscle strength, particularly when confounding factors like chronic inflammation exist. Future research should extend observation periods and incorporate body composition analysis with inflammatory marker assessments for comprehensive evaluation of nutritional interventions on muscle function.

No significant differences were observed between the two groups in frailty (FRAIL scale), cognitive function (MMSE), nutritional status (MNA-SF), depression (GDS-15), or quality of life (EQ-5D), suggesting limited impact of nutritional interventions (ONS or diet education) on multidimensional health in community-dwelling older adults with AA. The 3-month intervention duration may be insufficient to improve long-term cumulative indicators such as frailty or cognition, necessitating extended follow-up. Since ONS primarily targets caloric supplementation, future interventions should integrate exercise training or psychological support to address the multifactorial nature of geriatric frailty (e.g., mobility, mental health).

Several limitations of this study warrant discussion. As a non-randomized controlled trial, baseline imbalances persisted despite statistical adjustments, and the relatively small sample size necessitates cautious interpretation. Future multicenter studies with larger cohorts are needed to strengthen the evidence. The patient-driven intervention allocation (based on economic considerations and dietary preferences), while reflecting real-world practice, may introduce selection bias—an ideal design would employ double-blind placebo controls to minimize confounders. Methodologically, reliance on self-reported weight measurements introduced variability from transient factors like food intake and bowel movements. The study also lacked quantitative energy intake monitoring and objective parameters (e.g., inflammatory markers, body composition), limiting comprehensive metabolic assessment. Notably, existing evidence shows minimal ONS impact on long-term survival (20, 35), and our 3-month follow-up precluded evaluation of extended outcomes (e.g., mortality, functional status). Future work should extend observation to 6–12 months with laboratory and body composition measures. While we did not conduct formal nutrient composition comparisons between the two formulas (Ensure^®^ vs. Glucerna^®^) during the trial, our pre-study literature review found no significant differences in their macronutrient profiles. The variability in supplement composition could theoretically introduce intra-group heterogeneity, we need for future studies with larger samples to stratify outcomes by supplement type. Despite these limitations, as China’s first clinical trial investigating ONS for AA, these findings provide foundational data for larger studies. The identified preference patterns based on baseline characteristics offer valuable insights for personalized nutritional strategies.

## 5 Conclusion

Both ONS and diet education improved appetite in AA, with ONS demonstrating earlier efficacy. No intervention conferred significant functional or metabolic benefits within 12 weeks. SNAQ baseline strongly predicted outcomes (*β* = 0.566, *p* < 0.001), advocating personalized protocols for high-risk subgroups.

## Data Availability

The raw data supporting the conclusions of this article will be made available by the authors, without undue reservation.
